# Instantaneous Cure of Whooping Cough

**Published:** 1889-10

**Authors:** 


INSTANTANEOUS CURE OF WHOOPING-COUGH.—In the *Archievs of Pharmacy*, 1889, page 382, it is stated that the instantaneous cure of whooping-cough was attained by Dr. M. Mohn, as a result of accidentally observing that the disinfection of the sick-room of the whooping-cough patient by sulphurous acid caused the disappearance of the paroxysms with a rapidity bordering on the marvelous. The patients are freshly clad in the morning, and placed in another room, in which they remain during the day. Meanwhile, 25 gm. of sulphur is burned in the sick-room to each c. cm. of space; and after the bedclothing, garments, etc., have been properly spread out, and the sulphurous acid been permitted to permeate the air for five hours, the patients return to their disinfected sleeping-rooms in the evening, and are cured of whooping-cough.

Physicians may not generally be aware of the fact that sulphur bricks are obtainable, which may be burned to secure the effects of sulphurous acid by inhalation, or for general disinfectant purposes. Parke, Davis & Co. supply these, as well as a general line of disinfectants for household use, and will afford physicians all desired information concerning them, on request.

